# Doom and Boom on a Resilient Reef: Climate Change, Algal Overgrowth and Coral Recovery

**DOI:** 10.1371/journal.pone.0005239

**Published:** 2009-04-22

**Authors:** Guillermo Diaz-Pulido, Laurence J. McCook, Sophie Dove, Ray Berkelmans, George Roff, David I. Kline, Scarla Weeks, Richard D. Evans, David H. Williamson, Ove Hoegh-Guldberg

**Affiliations:** 1 Centre for Marine Studies and Australian Research Council Centre of Excellence for Coral Reef Studies, The University of Queensland, St Lucia, Brisbane, Queensland, Australia; 2 Great Barrier Reef Marine Park Authority, Townsville, Queensland, Australia; 3 Australian Research Council Centre of Excellence for Coral Reef Studies, Townsville, Queensland, Australia; 4 Australian Institute of Marine Science, Townsville, Queensland, Australia; 5 Centre for Marine Studies, The University of Queensland, St Lucia, Brisbane, Queensland, Australia; 6 Centre for Remote Sensing and Spatial Information Science, The University of Queensland, St Lucia, Brisbane, Queensland, Australia; 7 School of Marine and Tropical Biology and Australian Research Council Centre of Excellence for Coral Reef Studies, James Cook University, Townsville, Queensland, Australia; University of California San Diego, United States of America

## Abstract

**Background:**

Coral reefs around the world are experiencing large-scale degradation, largely due to global climate change, overfishing, diseases and eutrophication. Climate change models suggest increasing frequency and severity of warming-induced coral bleaching events, with consequent increases in coral mortality and algal overgrowth. Critically, the recovery of damaged reefs will depend on the reversibility of seaweed blooms, generally considered to depend on grazing of the seaweed, and replenishment of corals by larvae that successfully recruit to damaged reefs. These processes usually take years to decades to bring a reef back to coral dominance.

**Methodology/Principal Findings:**

In 2006, mass bleaching of corals on inshore reefs of the Great Barrier Reef caused high coral mortality. Here we show that this coral mortality was followed by an unprecedented bloom of a single species of unpalatable seaweed (*Lobophora variegata*), colonizing dead coral skeletons, but that corals on these reefs recovered dramatically, in less than a year. Unexpectedly, this rapid reversal did not involve reestablishment of corals by recruitment of coral larvae, as often assumed, but depended on several ecological mechanisms previously underestimated.

**Conclusions/Significance:**

These mechanisms of ecological recovery included rapid regeneration rates of remnant coral tissue, very high competitive ability of the corals allowing them to out-compete the seaweed, a natural seasonal decline in the particular species of dominant seaweed, and an effective marine protected area system. Our study provides a key example of the doom and boom of a highly resilient reef, and new insights into the variability and mechanisms of reef resilience under rapid climate change.

## Introduction

Coral reefs are among the most biologically diverse and economically important ecosystems. However, reefs are rapidly degrading at a global scale, due to a combination of pressures, including climate change, overexploitation, coral diseases, and declining water quality [1]–[4]. Rising ocean temperatures have triggered mass coral bleaching events that have devastated many coral reefs around the world [5] and caused ecological phase or state shifts, from coral-dominance to dominance by seaweeds (fleshy algae) [6]–[8]. Current climate change models suggest increasing frequency and severity of mass coral bleaching events [5], so that phase shifts to algal dominated states are expected to occur more frequently and last longer [9]–[11].

Critically, the recovery of degraded reefs depends on the reversibility of seaweed dominance [12], [13]. However, all previously documented cases have found dominance by seaweeds difficult to reverse, because the algae prevent settlement of new corals, and because the algae persist, usually due to overfishing or mass mortality of key herbivorous species and to relative unpalatability of algae to herbivores [14], [15]. Examples of natural reversals from algal dominance to coral dominated states are extremely rare (but see [16], [17]) and take years to decades to occur (e. g. Kaneohe Bay, Hawaii [18]; Dairy Bull Jamaica [19]). Rapid reversals from algal dominated states to dominance by corals and small algae have only been demonstrated at a very small scale after experimentally induced herbivore exclusion [20]. In that experiment, artificially enhanced algal biomass was rapidly consumed by grazers upon removal of exclusion cages, and reef recovery was dependent on recovery of herbivory, a process extrinsic to the corals and algae.

Inshore, high latitude coral reefs of the largest reef system in the world, the Great Barrier Reef (GBR), Australia, suffered severe mass bleaching of coral in early 2006. Reefs in the area exhibit low coral species diversity and are widely dominated by *Acropora* corals, with branching *Acropora* accounting for more than 90% of the coral species [21]. Sea surface temperatures in the inshore reefs of the Keppel Islands (23°10′S, 151°00′E) in the southern GBR rose rapidly in late 2005, with some locations reaching temperatures in December that are not normally found until February. The onset of high sea temperatures early in the season triggered coral bleaching by mid January 2006 [22]. Overall, bleaching damage was severe, affecting 77–95% of coral colonies [22], [23]. The purpose of this paper was to document some novel mechanisms for coral reef resilience based on changes in coral and seaweed abundance following the 2006 mass coral bleaching event that affected reefs of the Keppel Islands.

## Results and Discussion

**Figure 1:**
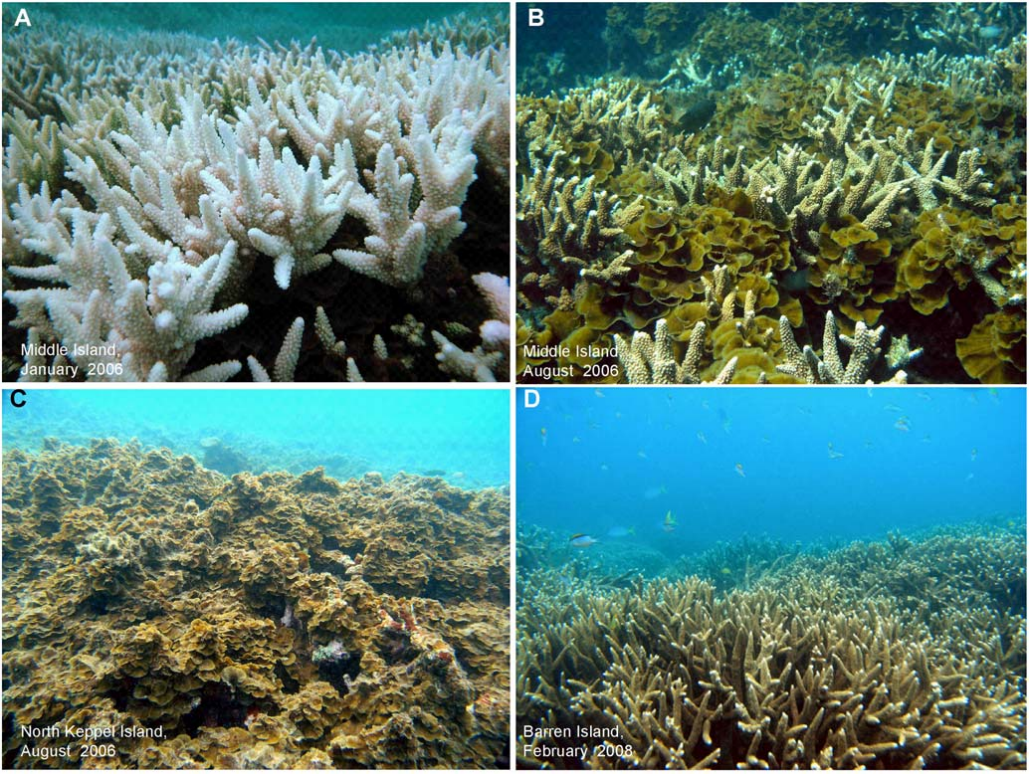
Coral bleaching, algal overgrowth of corals and coral recovery. A) Bleached corals in the Keppel Islands, Great Barrier Reef, during the mass bleaching event in January 2006. The fleshy brown seaweed *Lobophora variegata* grows at the base of the branches of *Acropora* spp. corals. B) *L. variegata* is released from space competition by coral mortality and overgrows coral skeletons as well as some coral tissue, causing an unprecedented algal bloom. C) Seaweed bloom on North Keppel Island after coral bleaching. The reef has lost its structural complexity and has experienced little coral recovery. D) Recovered reef on Barren Island, showing high coral cover and low cover of seaweeds.

**Figure 2:**
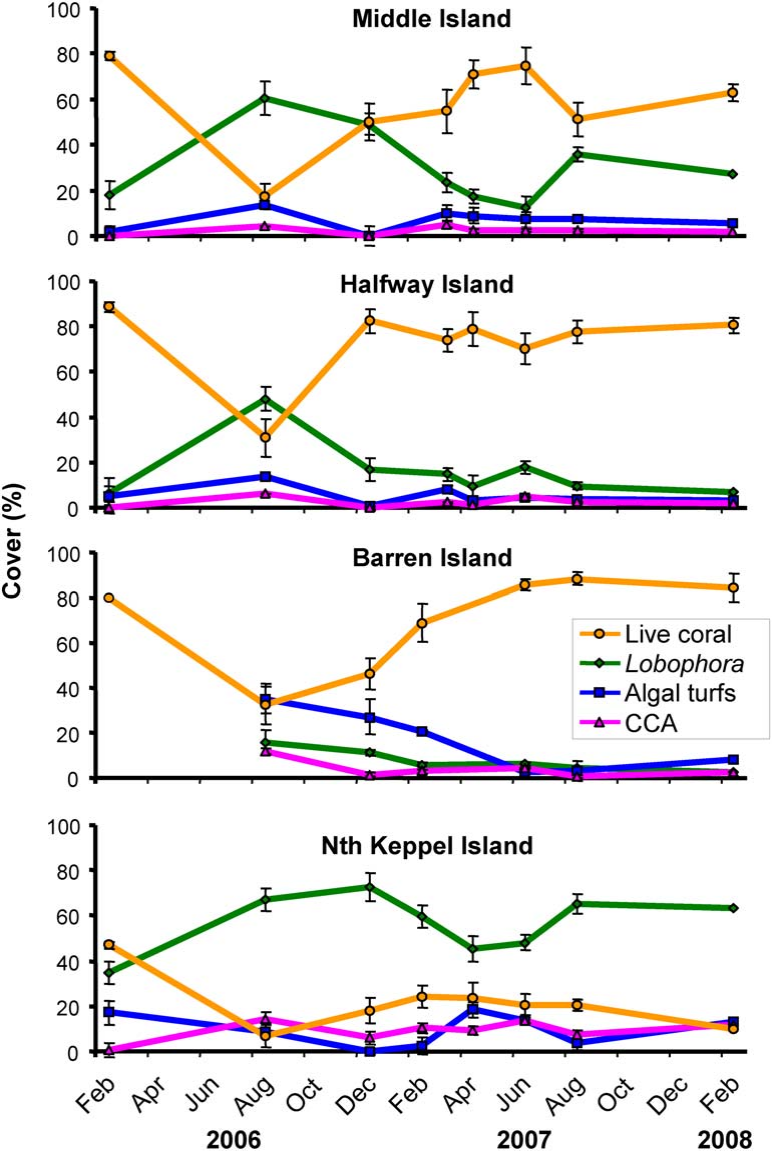
Coral – algal dynamics in response to the 2006 warming-induced coral bleaching event. Data from the reef slopes of four islands in the Keppel Islands, southern Great Barrier Reef. % cover data are means (n = 10) ±SE, except for Feb 2006 (n = 25–26). CCA: Crustose calcareous algae.

Abundance of corals and seaweeds showed strong dynamics in response to the warming-induced mass coral bleaching event (Figs. 1, 2). Cover of bleached but living coral (mainly branching *Acropora* spp.) on the reef slopes of Middle Island, Halfway Island, and Barren Island was high (77%–89%) during the bleaching event in January/February 2006. Five months after the onset of bleaching, coral cover was severely reduced, to values around 20–30% by July–August 2006. The coral mortality was followed by an extraordinary bloom of the brown seaweed *Lobophora variegata*, apparently unprecedented in magnitude on the GBR (GDP and LM personal observations, Fig. 2). This alga commonly grows between the branches of most *Acropora* colonies in the area, but under normal (i.e. undisturbed) conditions it is not able to grow beyond the base of the branches, probably due to competitive inhibition by the corals. Previous work on *L. variegata* growing amongst branching *Porites cylindrica* corals showed that the interaction is competitive, with both coral and alga inhibiting growth of the other [24], [25]. However, seaweeds and algal turfs were apparently released from space competition with the corals due to the bleaching mortality [9] and dramatically increased in cover (200–300% increase on Middle Island and Halfway Island) by August 2006. Importantly, coral bleaching preceded *L. variegata* overgrowth, and overgrowth only took place on bleached or dead corals at a range of spatial scales (from cm to 10 s of kilometers; careful inspection showed negligible overgrowth of healthy coral). Nonetheless, the seaweed apparently exacerbated coral mortality by overgrowing stressed coral tissue [24]–[26] (Figure S1D). Algal competitiveness may have been enhanced by uptake of nutrients and carbon generated by the coral mortality [27]. There are no previous observations of such an extensive bloom of *L. variegata*, or indeed any single species of fleshy alga, on the GBR, although large-scale blooms of filamentous algal turfs have occurred following coral mortality [9], [28], [29], and a small-scale bloom of a red seaweed was recorded in response to a ship-grounding [30]. Blooms of *L. variegata* are common in the Caribbean, particularly after the die-off of the sea urchin *Diadema* [14], [31] and following coral mortality [32], [33] (also personal observations in Islas del Rosario, Colombia and Flower Garden Banks, Gulf of Mexico, GDP and LM).

Surprisingly however, the cover of branching *Acropora* corals at most sites showed an extremely rapid recovery after the seaweed bloom, reaching pre-bleaching levels by December 2006–April 2007 (ca 12–14 months after the onset of bleaching, Fig. 2, Table 1). This represents a 100 to 200% increase in cover of *Acropora* in approximately 6 months, thereby returning the system to coral dominance (P = 0.004, 0.001 and 0.006 for Tukey's comparisons of August 2006 c.f. February/March 2007 for Middle, Halfway and Barren Islands respectively).

**Table 1:** Two-way analyses of variance for the effects of sampling date and site on % cover of corals, brown seaweed *Lobophora variegata*, algal turfs and crustose calcareous algae (CCA).

Source of variation	df	Mean-Square	F-ratio	p
**Coral cover**				
Date (D)	5	1.265	20.238	<0.001
Site (S)	3	5.257	84.100	<0.001
D×S	15	0.204	3.262	<0.001
Error	214	0.063		
***Lobophora*** ** cover**				
Date (D)	5	0.541	14.450	<0.001
Site (S)	3	5.249	140.224	<0.001
D×S	15	0.121	3.244	<0.001
Error	214	0.037		
**Algal turf cover**				
Date (D)	5	0.161	5.207	<0.001
Site (S)	3	0.096	3.094	0.028
D×S	15	0.184	5.929	<0.001
Error	214	0.031		
**CCA cover**				
Date (D)	5	0.203	15.964	<0.001
Site (S)	3	0.424	33.336	<0.001
D×S	15	0.018	1.378	0.160
Error	214	0.013		

Data were Arc-sin transformed. Interactions between date and site were significant; therefore, data were analysed for site effects within dates and date effects within sites, using a one-way ANOVAs and Tukey's post-hoc comparisons (results not shown).

**Figure 3:**
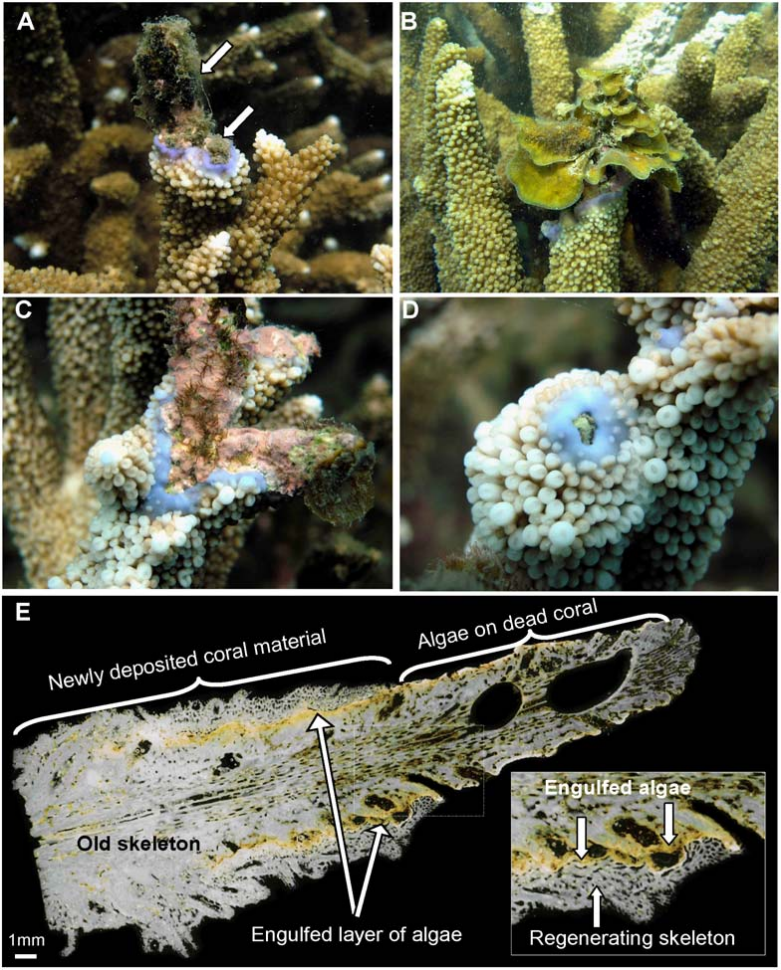
Coral recovery following algal overgrowth. Branches of *Acropora* corals died after bleaching and were subsequently colonized by a variety of benthic algae. Remnant coral tissue at the base of the coral colonies regrew upward and deposited new skeleton along the old dead coral branch, overgrowing A) algal turfs (arrows), B) fleshy seaweed *Lobophora variegata*, and C) crustose coralline algae. D) Coral tissue has all but completely overgrown the colonizing algae. E) Thin section of coral showing benthic algae sandwiched between old coral skeleton and a thin layer of new skeleton. Examination using a compound microscope showed that coral tissue overgrew a range of algal types.

**Figure 4:**
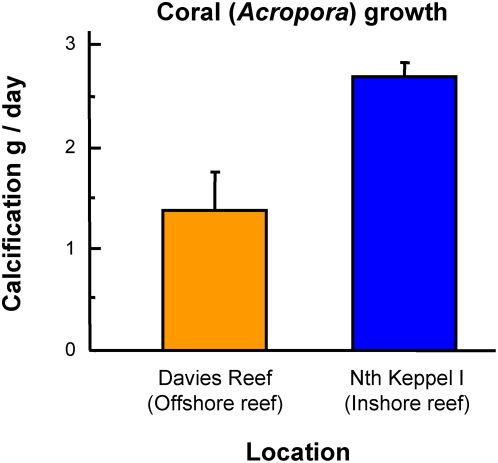
Coral growth (calcification). Calcification rates of *Acropora millepora* at North Keppel Island and Davies Reef (an offshore reef). Data are means±SE (n = 12 for North Keppel Island and 8 for Davies Reef), and show unusually high growth rates in the Keppel Islands.

Unexpectedly, the rapid reversal and increase in coral cover did not involve settlement and recruitment of coral larvae. Coral recruitment was generally very low throughout the course of the study at all sites [recruit densities for Middle, Halfway, Barren and North Keppel Islands were 0, <1, <1 and 4 recruits m^−2^ respectively; Kruskal-Wallis Test indicated no increases in recruit densities through time after the bleaching event, Table 2]. Instead, coral recovery involved a rapid regeneration and regrowth of remnant coral tissue after bleaching mortality, with branches of *Acropora* emerging from the algal mat to reestablish high cover much faster than could occur from growth of new recruits (Figs. 2, 3). Growth rates of branching *Acropora* from the Keppel Islands appear unusually high, with rates of calcification nearly 100% faster than those of corals from offshore the GBR (Fig. 4). Linear extension rates of branching *Acropora* from other Pacific inshore reefs are also extraordinarily high, with mean values of 333 (±42 SD) mm/year [34]. This rapid, vegetative regeneration allowed the corals to out-compete and overgrow the algae settled on dead skeletons.

**Table 2:** Kruskal-Wallis one-way analyses of variance for the effects of sampling date on density of coral recruits of four islands.

Source of variation	df	H	p
**Middle I**			
Date	5	0.000	1.000
**Halfway I**			
Date	5	5.000	0.416
**North Keppel I**			
Date	4	4.387	0.356
**Barren I**			
Date	5	11.308	0.046*

Data were log transformed. H: Statistic of the Kruskal-Wallis test; df: degrees of freedom. Dates included in the analyses are: Aug 06, Dec 06, Feb 07, Jun 07, Aug 07, Jan 08. Data missing for Dec 06 in North Keppel Island.

* Recruit density was slightly higher in February 2007 but declined afterwards.

We propose that this unusually rapid and successful regrowth stems from several key factors: i. the strong competitive ability of the corals; ii. the corals' ability to regrow from relatively small amounts of live tissue; iii. and a seasonal dieback in the single species of dominant seaweed. Although overgrowth by seaweeds probably inhibited coral growth, a natural seasonal decline in *L. variegata*, between December 2006 and March/April 2007 (Fig. 2), markedly reduced the apparent effects of this competitive inhibition. Cover of *L. variegata* decreased significantly from 50% to <20% in Middle Island and from 75% to 45% in North Keppel Island during that period of time (Table 1; P<0.005 for Tukey's comparisons of August 2006 and March/April 2007 for both islands).

**Figure 5:**
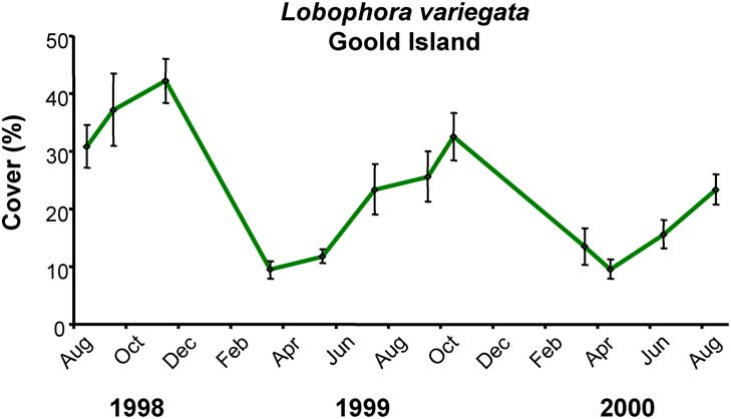
Seasonality in *Lobophora variegata* on Goold Island, inshore central GBR. Abundance of *L. variegata* consistently shows strong declines during the austral summer. Data are means±SE of 5 replicate quadrats.

**Figure 6:**
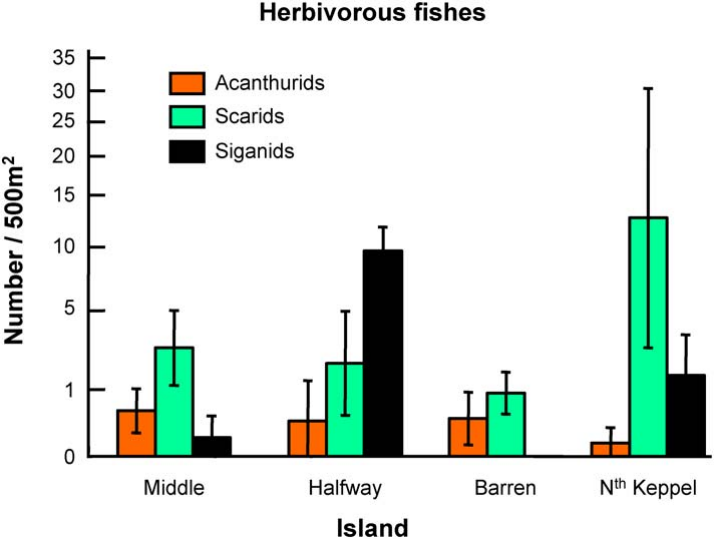
Herbivore abundance. Herbivore density data from the study sites; data are square root transformed, means+/−SE of 5 transects per site.

Removal of the seaweed *L. variegata* in this study appears to have been largely due to inherent seasonal dieback. Large amounts of loose *L. variegata* were observed at the time of the dieback, and similar seasonal changes in *L. variegata* have been previously observed in the GBR (Fig. 5) and nearby areas [35], apparently related to elevated seawater temperature during the austral spring and summer (GDP unpublished data). Herbivorous fishes, although largely unfished, are not generally abundant in the Keppel Islands, being generally about an order of magnitude less than on mid and outer shelf reefs [36]. Careful observations did not indicate grazing damage to the *L. variegata*, despite the extent of the bloom and decline, and patterns of herbivore abundance among the study reefs were not consistent with the growth and decline in *L. variegata* at these sites (Fig. 6). The site with lowest herbivore densities had lowest *L. variegata* abundance (Barren Island). The site with most abundant scarids had most abundant *L. variegata* (North Keppel Island), while siganids were most abundant on Halfway Island, which had intermediate abundance of *L. variegata*. Large invertebrate herbivores, such as sea urchins, were virtually absent across all sites. Thus, whilst herbivory could have contributed to some degree, and is likely important to algal abundance on these reefs generally, the extent of decline in *L. variegata* in this study appears largely due to seasonality.

However, the increase in coral cover was apparently also due to strong growth rates and consequent competitive ability of the coral, and not dependent on the seasonal decline in the algal competitor, *L. variegata*. This is suggested by results for Middle Island (Fig. 2; August–December 2006) where strong coral recovery preceded decline in *L. variegata*, and from Barren Island, where coral recovery involved overgrowth of non-seasonal algal turfs and crustose calcareous algae. Tissue growth may have been enhanced by heterotrophic feeding [37], as shown elsewhere on GBR reefs [38].

Regeneration of the coral tissue apparently derived from tissue reservoirs, or areas of live coral tissue that persisted at the very base of the coral branches, underneath the seaweed canopy (Figure S1A, B; the “phoenix effect” in which apparently dead coral branches regenerate live tissue [39]–[41]). Removal of the dominant seaweed mat showed that coral tissue mortality was extensive under the seaweed at all sites. However, there did remain small fragments of live coral tissue. The remnant surviving coral tissue rapidly expanded upwards along the dead coral branches (Fig. 3) and actively overgrew *L. variegata*, as well as a range of other algal types, including filamentous algal turfs, fleshy seaweeds and crustose coralline algae (Fig. 3A–D). Thin sections of *Acropora* corals show overgrowth of several algae by new coral material, and show that overgrowth involved direct horizontal contact as well as overtopping, resulting in a “seaweed sandwich [42]”, with algae engulfed between new and old layers of skeleton (Fig. 3E). Regeneration over existing coral skeletons offers an energetically efficient and rapid mechanism for recovery, by limiting the calcification required for regrowth. Whilst regeneration of corals has been observed elsewhere [42], [43], our findings are significant because they demonstrate the potential importance of this process for large-scale, rapid recovery even after severe climate-related mass bleaching. The rate and scale of recovery is increasingly critical as climate change causes more frequent mass bleaching events.

Coral recovery and algal dynamics were not uniform in this study. Although most reefs showed rapid recovery, coral cover on North Keppel Island declined from 46% to <10% after bleaching and had recovered relatively little after two years (Fig. 2), despite a marked seasonal decline in *L. variegata*. Coral cover on North Keppel Island prior to the bleaching event was low compared to the other reefs in the area (46% vs. 75–90% respectively) and cover of *L. variegata* higher. These differences may reflect differences in disturbance history, conditions less conducive to coral growth, or differences in the extent of coral mortality due to floods from the Fitzroy River (the largest river catchment along the GBR) [44]. Recovery of the reef on North Keppel Island may also have been limited by the loss of three-dimensional structure of the reef framework (most branching *Acropora* corals have been broken into rubble due to bioerosion, Fig. 1C; habitat complexity has been shown to be critical for the rapid recovery of damaged reefs [19], [45]).

At the other extreme, coral recovery at Barren Island was very strong, and abundance of *L. variegata* remained much lower than other sites, even after coral mortality (Fig. 2). However, abundance of *L. variegata* was still highest following coral mortality (18%), and declined as the coral recovered (although not significantly: P = 0.131 for Tukey's comparison of August 2006 and February 2007). Barren Island is further offshore and in deeper water than the other sites, and dead coral tissue was colonized predominantly by algal turfs more typical of offshore reefs. Detailed analyses of the species composition of the algal turfs in this locality (data not shown) revealed a very different species composition of turfing algae, mainly dominated by calcareous turfing species (e.g. *Jania* and *Amphiroa*).

Recent events in the Keppel Islands provide an exceptional, but important example of the doom and boom of highly resilient reefs, and thereby provide new insight into the potential variability in mechanisms of reef resilience. Most degraded reefs globally have either failed to recover from events such as coral bleaching and other human induced disturbances [3], or have taken several years to decades to return to pre-disturbance condition [14], [15], [18], [19], [29], [46], [47]. In contrast, the Keppel Islands have shown rapid recovery of coral dominance, despite repeated coral bleaching events (1998, 2002, and 2006 [48]), severe flood plumes (e.g. 1991, 2008 [44]), and dense algal overgrowth. If they allow recovery of coral populations within one year, instead of ten, such exceptional processes may be disproportionately important to larger-scale reef resilience.

Resilience of reef coral populations is typically considered in terms of removal of algal blooms by herbivores, combined with replenishment by coral larvae. Whilst these factors are no doubt vital for reef persistence [7], [13], [49], both abundance of herbivorous fishes and coral recruitment were apparently limited on the reef slopes studied here during these events. There is considerable evidence that algal abundance on coral reefs is generally related to herbivory [6], [7], [50]–[54], and herbivory can be important to interactions between *L. variegata* and corals on the GBR [25]. However, in this instance, removal of the seaweed *L. variegata* appears to have been largely due to inherent seasonal dieback, more than consumption by herbivores, although experimental studies would be required to be conclusive. Importantly, this dieback is apparently species specific ([35], GDP unpublished data), so that its ecological significance presumably depends on the nature of the seaweed bloom as a single species. In more typical multi-species seaweed blooms, it is unlikely that all species would have similar seasonality, and competitive effects on coral regrowth would probably be stronger. In this sense, given the apparent limited abundance of herbivores, the reduction in seaweed during our study may be a fortunate coincidence of monospecific bloom and seasonal dieback in that one species. Further, had the decline in *L. variegata* not coincided with rapid coral growth, it is likely that a range of other algae would have colonized, potentially stabilizing the phase shift. Thus, the seasonal decline was clearly important to the resilience of these reefs in these circumstances, but should not be seen as diminishing the general importance of herbivory to reef resilience.

Our results stand in contrast with many previous studies, especially studies of coral and algal dynamics on Caribbean reefs in the early 80 s, where a combination of coral mortality and hurricane damage followed by mortality of sea urchins, caused massive algal blooms (including *L. variegata*) that still continue today [14], [15], [55]. Although *L. variegata* was involved in both circumstances, there are several fundamental differences that probably contribute to the different outcomes. First, the Keppel Islands are dominated by rapidly growing, branching *Acropora*, apparently better suited to competing with a mat-like algal growth than the massive and plate-like corals that were dominant on Caribbean reefs [26], [55], [56]. Coral-algal interactions will depend considerably on the particular species involved. Second, the monospecific algal bloom in the Keppel Islands was exceptionally vulnerable; most macroalgal blooms are much more diverse, imbuing the algal-dominated state with greater resilience. Studies of Caribbean reefs typically note 5–10 genera of benthic macroalgae (e.g. [31], [57], [58]); after long-term herbivore exclusion on the GBR, at least 10 algal genera were abundant in algal dominated plots [7].

Third, coral recovery may be strongly influenced by the nature of the disturbance regime. Reefs subject to acute disturbances, such as the rapid bleaching in the Keppel Islands, may often recover more effectively than those subject to chronic disturbances such as in the Caribbean [46], [59]. Similarly, the spatial scale of disturbance in our study was much smaller than that in the Caribbean. Numerous other factors can contribute to the resilience or vulnerability of a reef (e.g. [3], [60]).

In summary, unusually rapid coral recovery in the Keppel Islands apparently stemmed from synergistic effects of factors not previously recognized as important to resilience. These factors included robust tissue regeneration, high competitive ability of the corals and a seasonal dieback in the monospecific seaweed bloom, all against a backdrop of an effective marine protected area system and moderate water quality. Understanding the variability in mechanisms underlying resilience is critical for reef management under climate change. Settlement and recruitment of new corals requires years to decades to re-establish abundant corals, whereas recovery in the Keppel Islands took less than one year. Frequent, large-scale damage may mean that reefs able to rapidly recover abundant corals may serve as key refugia, or sources of larvae for reef recovery at broader scales. Diversity in processes may well be critical to the overall resilience and persistence of coral reef ecosystems globally.

## Materials and Methods

We monitored the dynamics of corals and benthic algae on the reef slopes (4–7 m depth) of four islands [Middle (North side, Surprise Rock: 23°09.896 S; 150°55.420 E), Halfway (Southwest side: 23°12.193 S; 150°58.187 E), North Keppel (Southeast side: 23°05.123 S; 150°53.983 E) and Barren (South East side, Coral Gardens: 23°09.796 S; 151°05.507 E)]. On each reef slope, the % cover of corals (using functional forms, e.g. branching, massive, mushroom, and genera) and benthic algae (functional forms and genera) was quantified in an area of ca 20 m×4 m using 10, 50×50 cm randomly allocated quadrats (with 10×10 grids) in August and December 2006, March, April, June and August 2007, and February 2008. The number of coral recruits (colonies <5 cm diameter) in each quadrat was also scored. % cover of benthic organisms during the onset of the bleaching event (January–February 2006) was estimated from 25–26 photo-quadrats (1×1.3 m) along 50 m transects. Cover of bleached coral on Barren Island in January 2006 was estimated visually and from an aerial photograph (projected onto a grid of 100 quadrats, with each quadrat scored for bleaching). Although different methods were used to quantify corals and algae during the onset of the bleaching (first sample date) and the rest of the sampling dates (7 dates), the extent of any differences due to methods are likely to be minor compared to the differences between dates due to ecological changes.

Cover and coral recruitment data were analyzed for differences between sampling dates and sites using a two-way analysis of variance, with dates and localities as fixed factors and quadrats as replicates. Data were checked for normality using stem and leaf plots and probability plots; and for homogeneity of variances with Cochran's test. Coral recruitment data were analyzed using a non-parametric Kruskal-Wallis test.

Coral growth data (represented by calcification rate) was determined at North Keppel Island and, for comparison, at an offshore reef in the central GBR (Davies Reef, 18.8°S, 147.6°E). 8 to 12 colonies (15–20 cm size) of branching *Acropora millepora* were collected at each location, wet weighed on land and returned to the water. The experiment was set up in February 2003 and the corals reweighed in June and September 2003. Although less accurate than the buoyant-weight method [61], the wet-weight method is adequate for estimating relative and gross differences between locations over a relatively long time frame (7 months).

Seasonality in *L. variegata* was also measured on Goold Island on the inshore, central Great Barrier Reef (18°10.9′S, 146°10.2′E) from 1998 to 2000. Cover was estimated using 5 replicate, fixed 50×50 cm quadrats randomly located on the reef flat.

Density of herbivorous fish was measured using underwater visual census by scuba. Five 50 m×10 m (500 m^2^) replicate belt transects were censused at each site in March 2007. Transects were laid haphazardly along the reef slope [62].

Coral regrowth was examined using thin sections prepared from *Acropora* branches (10–15 cm), air-dried and then sectioned in the laboratory, using a bench saw. Longitudinal and latitudinal sections of regenerating axial branches were cut into slices approximately 5 mm thick (n = 10). Select sections were prepared for thin sectioning and fixed in Epoxicure resin (Buehler Ltd, Lake Bluff, IL, USA) onto 50 mm×76 mm slides. Sections were polished to an approximate thickness of 25 µm and analysed under light microscopy.

## Supporting Information

Figure S1 Coral mortality and tissue remnants following coral bleaching. A) Dead *Acropora* sp. colony colonized by algal turfs and with *Lobophora variegata* seaweed at the base. B) Dead *Acropora* colony with part of the *L. variegata* canopy removed, showing remnant pigmented coral tissue (inset). C) *L. variegata* overgrowing *Acropora* corals. D) Identical to C) but with the algae removed, showing variable localized bleaching of live coral tissue and some coral mortality occurring underneath the algal canopy. Live coral tissue at the base of the branches may act as tissue reservoirs for future rapid coral recovery.
(9.97 MB TIF)
