# Supplementary figures and images for: Doom and Boom on a Resilient Reef: Climate Change, Algal Overgrowth and Coral Recovery
Source: PLoS One. 2009 Apr 22;4(4):e5239. doi: 10.1371/journal.pone.0005239 (PMC2668766; doi:10.1371/journal.pone.0005239)

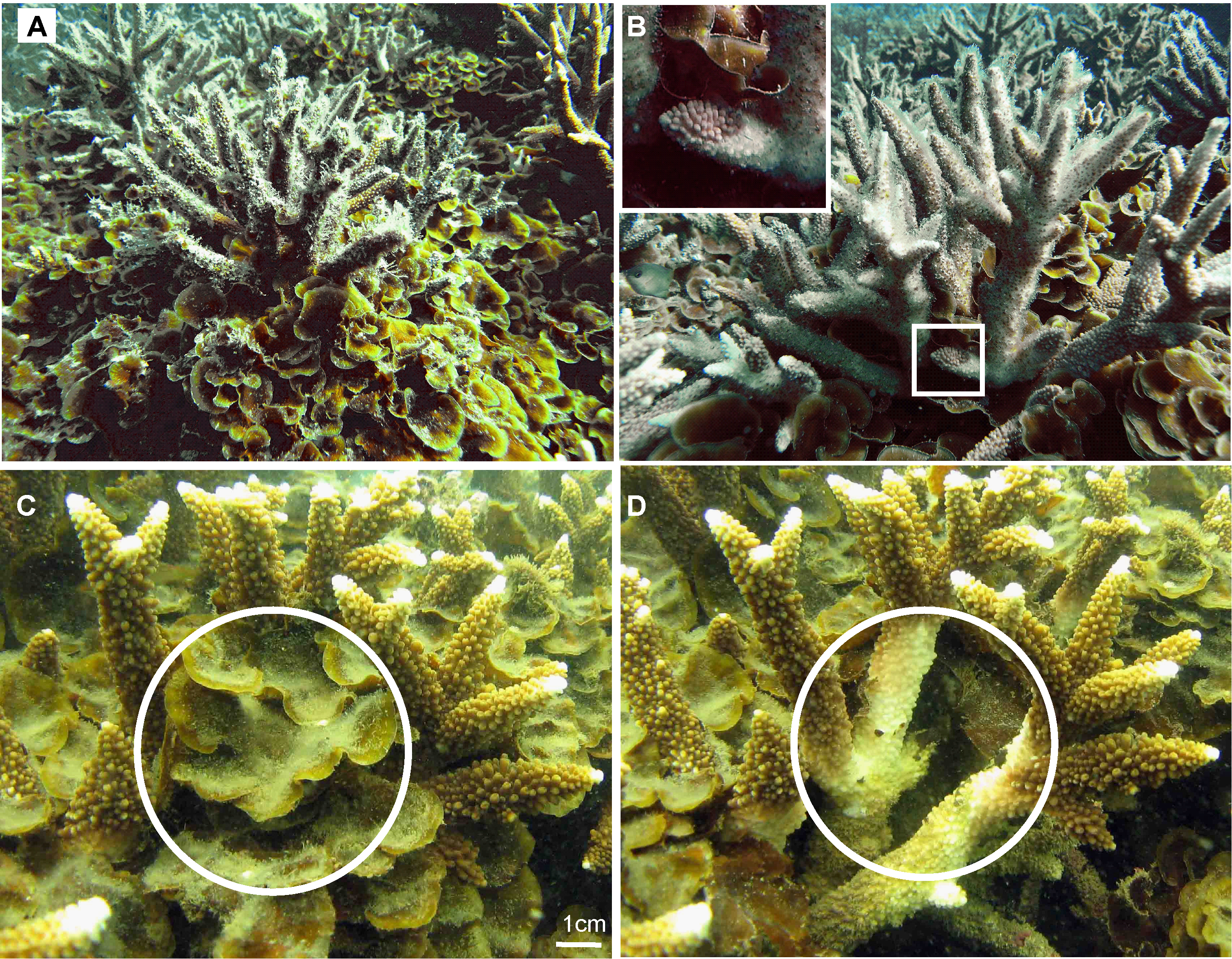

Supplement: Figure S1 — Coral mortality and tissue remnants following coral bleaching. A) Dead Acropora sp. colony colonized by algal turfs and with Lobophora variegata seaweed at the base. B) Dead Acropora colony with part of the L. variegata canopy removed, showing remnant pigmented coral tissue (inset). C) L. variegata overgrowing Acropora corals. D) Identical to C) but with the algae removed, showing variable localized bleaching of live coral tissue and some coral mortality occurring underneath the algal canopy. Live coral tissue at the base of the branches may act as tissue reservoirs for future rapid coral recovery. (9.97 MB TIF) [file pone.0005239.s001.tif]
